# Notes of the Week

**Published:** 1921-07-02

**Authors:** 


### NOTES OF THE WEEK.

### A CHANCE FOR A JOINT APPEAL.

How great a disappointment the grant of only half the amount recommended by Lord Cave's Committee has proved to be can be judged by the fact that in the provinces rumour put the probable recommendation as high as three million. Since the Government was perhaps never likely to grant the whole of any sum recommended, it might have been wiser to have suggested a larger sum, especially since a million was obviously a very modest estimate of the need. At all events the way is open for voluntary effort to raise a similar sum, so that the million recommended may be available. If a concerted appeal is to be made, here is the occasion and the excuse to make it. The practical point is this: Now the hospitals have a definite assessment of their need, to which the Government will only supply one half, can they invent a joint plan of campaign to raise the remainder? The force of public opinion at all events would be behind such an appeal, if the willingness and capacity are there to present and enforce it.

### ALEXANDRA DAY.

The sale of roses in the streets on Alexandra Day continues to be a most popular and successful event, and is not in any way affected by the slump that is attached to the ordinary flag-day collections. As usual, the Queen-Mother made her accustomed drive through the City and West End on the occasion, which was the tenth anniversary of the founding of this ingenious appeal for the support of hospitals and other charities. Her Majesty was accompanied by Princess Victoria, and was greeted everywhere by cheers and salutations of homage and respect. Over 12,000 ladies gave their services in connection with the event, and from early morning until twilight the attractive artificial flowers were on sale at the railway termini and throughout the streets. A particular request was that the collection should be a silver one, and certainly silver appeared to be passed into the boxes as freely as copper. In some districts, of course, Treasury notes also were forthcoming. At Christie's, the well-known auctioneers, where that day there was a sale of some remarkable sapphires, a bouquet supplied by Queen Alexandra was put up for sale, and was returned to be sold at least forty times, procuring during the process a contribution to the fund of £1,141.

### THE SATURDAY FUNDS AND TRADE DEPRESSION.

Notwithstanding the severe depression of trade, the first returns of the Birmingham Hospital Saturday Fund reached some £43,500, or £600 more than at the same stage of the collection in 1920. The number of contributors has grown in the past year by 202, and by the time that this appears every reason suggests that the grand total of £60,000 attained last year will have been reached or surpassed. The indications are gratifying, not only in themselves, but because they suggest that, when more normal conditions are restored to industry, the Saturday Funds may prove a steadily increasing source of regular income. In other words, the impetus given by the war seems to be surviving the reaction which has inevitably followed. In 1922 the Birmingham Hospital Saturday Fund will have reached its jubilee, and the traditional goal of £100,000 may be coming within reach. Everything points to our reiterated suggestion that the solution of the voluntary hospital finance lies in the extension of the principle of weekly collections from works and factories to shops and offices generally. The latter are, roughly, in the elementary condition of organisation in which the factory collections were fifty years ago.

### HOSPITAL SUNDAY.

The weather in London on Hospital Sunday may be described as propitious. There was a storm in the night which must have unsettled the plans of most people who were minded to spend the day in the country, and shortly before church-time on Sunday evening there came a sudden downpour calculated to send many in the direction of places of worship rather than to the parks. We noticed one or two collectors outside the Ritz and elsewhere, but the general impression was that there were not so many street helpers as last year. What effect the announcement that the Government had decided to recommend a grant of £500,000 had on collections is the subject of diverse opinions. Some think that the news did harm to Hospital Sunday, and others that it did good in view of the plain hint that public help to make up the other half-million is increasingly necessary. Some excellent donations have reached the Fund, including one of £5,000 from Lady Strathcona, and it is confidently hoped that last year's collection of £121,000 has been exceeded. Viscount Goschen, Treasurer of Guy's Hospital, told the congregation of St. Martin's-in-the-Fields that 2*s*. per head per year from the inhabitants of Greater London would put the metropolitan hospitals on their feet.

### THE HOSPITAL PAGEANT.

A further meeting of hospital representatives to consider the replies of the three Funds to the resolution of May 20 will be held on Friday, July 15, at 3 p.m., in the Board Room of the National Hospital for the Paralysed and Epileptic, Queen Square, W.C., by kind permission of the Board. The meeting will also consider the advisability of approaching the Committees of all the London hospitals, and the appointment of an Executive Committee to prepare a definite scheme. We understand that the replies show the Funds to be favourably disposed and that a large meeting on the 15th is anticipated.

### LONDON SECRETARYSHIPS.

Mr. J. P. Wetenhall, B.A., Oxon, has been appointed secretary to the West End Hospital for Nervous Diseases, in succession to Mr. D. Kirkaldy-Willis, who has retired. We learn that the appointment of Mr. Inman to Charing Cross has now been confirmed, and that he will take up his duties there in October, remaining with the Saturday Fund till the end of September.

### MAINTENANCE CHARGES DEFERRED.

Once again the question of maintenance charges from patients at the General Hospital, Birmingham, has been deferred in deference to the wishes of the Saturday Fund and the subscribing trade unionists. The hospital, however, is £25,000 a year in debt, and the suggestion is made that while the manual and factory workers subscribe weekly, the shop assistants and commercial classes do not. If this is so, Birmingham and London seem to be parallel instances, for there can be little doubt that it is because London is rather commercial than industrial in the factory sense that so much difficulty is found in organising weekly contributions on a large scale. Bishop Hamilton Baynes has suggested that if the Oxford plan were adopted and a maintenance charge made only on those not at present subscribing the objection of the working-class subscribers to the maintenance charge would be withdrawn. A considerable sum is obtained at present from in-patients' voluntary payments, and it is suggested that if a more concentrated effort in this direction were made, coupled with a system of collection from out-patients attending the more costly special departments, such as *x*-rays, etc., a valuable addition to the income would result. Meantime it has been decided to reduce the beds at the Jaffray Branch Hospital at Erdington from sixty to thirty, a number which the income should be able to maintain.

### ENCOURAGEMENT FOR MATERNITY HOSPITALS.

While the Royal Victoria Infirmary, Newcastle, is in serious financial straits, the Newcastle Maternity Hospital has paid its way, and the corporation is about to begin the new wing which will add twenty beds to the hospital. Toward this the hospital is to provide one-quarter and the corporation three-quarters of the cost. It is, indeed, announced that the city will take over the hospital shortly. The ante-natal department had proved a success, and Dr. Lyle said that they were working in harmony with the corporation. Maternity hospitals, once neglected, were coming into their own and receiving support from the Government, councils, and public bodies. Maternity cases were increasingly going into institutions, where better results could be obtained. They were working the hospital as a voluntary institution until the time when the city might feel able to take it over.

### CONVALESCENCE IN LODGINGS.

The Liverpool Convalescent Institution, Wootton, has been feeling not only a want of financial support, but a lack of patients. Seeking to explain this, the Hon. Edward H. Cozens-Hardy, who is chairman of the council, suggests that many of the class from which the patients were drawn before the war were able till the end of last year to afford convalescence in lodgings. We should have supposed that such patients would have found the convalescent home more exhilarating, but the fact remains that nearly 100 more patients could have been accommodated. The chairman's suggestion that the admission of convalescents at an earlier stage might be a useful development, even though the nursing staff would thereby have to be increased, was welcomed by Mr. Wade Deacon, who remarked that the plan would relieve the present congestion in the city hospitals. We are glad to see that few public meetings of hospitals are allowed to pass without reference to the claim of the hospitals upon the surplus funds of the Approved Societies. If this policy is maintained, the tendency of the Societies to recognise the claim will be strengthened and a debt long overdue will be paid.

### A PATIENTS' LETTER-BOOK.

Is any systematic attempt made to file or use the letters of appreciation which regularly arrive at hospitals? That the publication of these in newspapers is useful, as well as general, is shown by the recent experience of the Coventry and Warwickshire Hospital. The receipt of a letter of thanks, accompanied by a gift of five pounds, called attention to a similar letter, lately published in a Midland newspaper, which had, it was said, served to refute some adverse criticisms. The meeting at which these letters were discussed resolved that these appreciations should be "collected and tabulated." In other words, a Patients' Letter-book is to be formed. We hope that the idea is not a new one. If these appreciations were more particular and less general in their phrasing than they usually are, they would be better worth preservation; but, even so, they contain hints of which use can be made. A certain London hospital is accustomed to dig in its own archives for the ideas on which its autumn leaflet of appeal is based. It has never tried, we think, a Patients' Letter-book. But where such a volume is kept, hints can be found.

### HOSPITAL STAMPS AT LEICESTER.

Envelopes sent out from the Leicester Royal Infirmary now bear a large blue stamp with a numismatic design upon it. Below the design is stamped "Leicester Royal Infirmary: 150th anniversary, September 11th, 1921." Beneath the hospital stamp is written: "If you like the artistic design of this stamp please secure a book of twelve, price 1s. 6d., or on cards of sixty, price 7s. 6d." Hitherto these stamps have had a greater popularity abroad, but we hope that this latest resort to them will be successful.

### GUARDIANS AND THE MIDDLE CLASSES.

The Ipswich Board of Guardians have decided to allot part of the Heathfield Institution to private patients at a charge of two and a-half guineas a week. There will be accommodation for twelve men and twelve women, who will be attended by a special nursing staff, and, if they wish, by their own doctor. The block allotted to this purpose will be known as the Heathfield Nursing Home, and at the end of three months the fee will be reconsidered in the hope that it may be possible to reduce it.

